# Systematic reviews need to consider applicability to disadvantaged populations: inter-rater agreement for a health equity plausibility algorithm

**DOI:** 10.1186/1471-2288-12-187

**Published:** 2012-12-19

**Authors:** Vivian Welch, Kevin Brand, Elizabeth Kristjansson, Janet Smylie, George Wells, Peter Tugwell

**Affiliations:** 1 Clinical Epidemiology Unit, Ottawa Hospital Research Institute, Ottawa Hospital, Ottawa, ON, Canada; 2 Centre for Global Health, Institute of Population Health, University of Ottawa, 1 Stewart Street, Ottawa, ON, K1N6N5, Canada; 3 Telfer School of Management, University of Ottawa, Ottawa, ON, Canada; 4 School of Psychology, Centre for Global Health, Institute of Population Health, University of Ottawa, 1 Stewart Street, Ottawa, ON, K1N6N5, Canada; 5 Centre for Research on Inner City Health, Keenan Research Centre of the Li Ka Shing Knowledge Institute, St. Michael’s Hospital, Toronto, ON, Canada; 6 Dalla Lana School of Public Health, University of Toronto, Toronto, ON, Canada; 7 Department of Epidemiology and Community Medicine and Department of Medicine, University of Ottawa, Ottawa, ON, Canada; 8 University of Ottawa Heart Institute, Ottawa, ON, Canada; 9 Clinical Epidemiology Unit, Ottawa Hospital Research Institute, Ottawa Hospital, Ottawa, ON, Canada; 10 Department of Epidemiology and Community Medicine and Department of Medicine, University of Ottawa, Ottawa, ON, Canada; 11 Clinical Epidemiology Unit, Ottawa Hospital Research Institute Ottawa Hospital, Ottawa, ON, Canada; 12 Centre for Global Health, Institute of Population Health, University of Ottawa, 1 Stewart Street, Ottawa, ON, K1N6N5, Canada

**Keywords:** Systematic reviews, Applicability, Health equity, Sex and gender, Socioeconomic status

## Abstract

**Background:**

Systematic reviews have been challenged to consider effects on disadvantaged groups. A priori specification of subgroup analyses is recommended to increase the credibility of these analyses. This study aimed to develop and assess inter-rater agreement for an algorithm for systematic review authors to predict whether differences in effect measures are likely for disadvantaged populations relative to advantaged populations (only relative effect measures were addressed).

**Methods:**

A health equity plausibility algorithm was developed using clinimetric methods with three items based on literature review, key informant interviews and methodology studies. The three items dealt with the plausibility of differences in relative effects across sex or socioeconomic status (SES) due to: 1) patient characteristics; 2) intervention delivery (i.e., implementation); and 3) comparators. Thirty-five respondents (consisting of clinicians, methodologists and research users) assessed the likelihood of differences across sex and SES for ten systematic reviews with these questions. We assessed inter-rater reliability using Fleiss multi-rater kappa.

**Results:**

The proportion agreement was 66% for patient characteristics (95% confidence interval: 61%-71%), 67% for intervention delivery (95% confidence interval: 62% to 72%) and 55% for the comparator (95% confidence interval: 50% to 60%). Inter-rater kappa, assessed with Fleiss kappa, ranged from 0 to 0.199, representing very low agreement beyond chance.

**Conclusions:**

Users of systematic reviews rated that important differences in relative effects across sex and socioeconomic status were plausible for a range of individual and population-level interventions. However, there was very low inter-rater agreement for these assessments. There is an unmet need for discussion of plausibility of differential effects in systematic reviews. Increased consideration of external validity and applicability to different populations and settings is warranted in systematic reviews to meet this need.

## Background

Health inequity is defined as avoidable and unfair differences in health [1]. Health inequity is caused by multiple, interacting factors described by the WHO Commission on Social Determinants of Health (CSDH) as 1) the socioeconomic and political context, 2) social position, 3) material circumstances and 4) the health system [2]. The health system described by the WHO CSDH is a broad concept which includes factors such as access to health care as well as the effectiveness of health care as well as public health and non-health interventions. Health care, health policy and non-health (e.g. social or financial) interventions may inadvertently increase health inequity if they are less effective in disadvantaged populations due to either prognostic factors (e.g. comorbidities or nutritional deficiencies) or treatment-covariate interactions (e.g. crowded home environment may increase transmission of infectious diseases, low literacy affects ability to benefit from written materials) [3].

Systematic reviews are useful as a basis for evidence-informed policy and practice [4,5]. Systematic reviews represent an opportunity to identify what works to promote health equity because they include studies conducted in a diversity of settings and populations allowing both prognostic factors and treatment-covariate interactions to be explored [6-8]. However, systematic reviews rarely assess whether interventions have intended or unintended effects on health equity. For example, only 1% of a random sample of Cochrane reviews assessed differences in effectiveness of interventions across socioeconomic or demographic factors [9]. Decision-makers such as NICE (National Institute of Clinical Excellence) in the UK and CADTH (Canadian Agency for Drugs and Technology in Health) in Canada are asking systematic reviews to assess the evidence of differential effects across age, sex and socioeconomic status. Failure to assess or consider effects on health equity in systematic reviews may lead to rejection of systematic reviews as a useful source of evidence for policy-makers who seek information on distribution of effects in the population [10,11], or may even lead to implementation of policies and programs which inadvertently increase health inequities [12,13].

Health inequity can be categorized using the PROGRESS-Plus framework –this is an acronym which summarizes factors across which differences in health may be considered inequitable depending on the setting and context: Place of residence; Race/ethnicity/culture; Occupation; Gender; Religion; Education; Socioeconomic status; Social capital [14]. There are additional personal characteristics (e.g. age, disability and sexual orientation) [15,16] that may present barriers to use of health and other public services; some of which are included in antidiscrimination laws. Furthermore, additional details may be helpful in describing vulnerable people depending on the focus of the study. For example, for studies of young people, additional characteristics related to vulnerability may include exclusion from school, looked after children or runaways. The term PROGRESS-Plus was coined to be a more inclusive framework for identifying both broad social and economic determinants of health (PROGRESS) and other characteristics, context and setting that are particularly relevant to the focus of study (Plus) [15]. Promoting health equity remains high on the agenda of local, national and international policy agendas [17].

Systematic reviews have been challenged to consider whether disadvantaged groups will obtain the same or more benefit than the mean effect, while at the same time minimizing the risk of spurious results due to multiple comparisons[12]. One way to reduce the chances of spurious results and increase the credibility of subgroup analyses is to specify comparisons prior to analysis [18-20] . Different authors are likely to argue *pre-hoc* for different groups. Thus, there is a need to assess how often there is disagreement amongst authors, and if so, develop guidelines for making these judgments.

This study aimed to develop and evaluate an algorithm to assess the likelihood of differences in relative effects of interventions in disadvantaged populations (across sex and socioeconomic status) relative to advantaged populations.

## Methods

The health equity plausibility algorithm was developed using steps based on Feinstein [21] and Streiner and Norman [22].

### Ethics approval

This research study was approved by the University of Ottawa Research Ethics Board (ethics approval certificate #H02-09-11b and #H02-09-11c).

### Purpose

The purpose of the health equity plausibility algorithm is to assess the likelihood of differences in relative effect of an intervention across population characteristics defined by PROGRESS-Plus. For example, aspirin reduces the risk of stroke in women but not in men [23]. As another example, the measles antibody response in children is lower among the malnourished [23]. We chose to focus on only two PROGRESS-Plus characteristics because we felt that this would be a difficult task for respondents, and multiple characteristics might lead to lack of attention to additional characteristics. We selected sex and socioeconomic status because they are amongst the most commonly reported differences in effects and burden of disease in the literature, and we felt respondents would feel the most able to make decisions about these characteristics. We recognize that differences across other characteristics such as ethnicity, educational attainment and occupation are important but these have not been assessed in this study.

It is important to note that the health equity plausibility algorithm is intended to predict likelihood of differences in relative but not absolute effects. Differences in absolute effects can be artifacts of differences in baseline risk, with no attendant difference in actual effect. For example, immigrants and refugees have a lifetime risk of developing active tuberculosis of over 35%. Assuming a relative risk reduction with isoniazid treatment of 93%, treating all immigrants and refugees will reduce lifetime cases of active tuberculosis by 33 in 100 people. In contrast, the typical high income country-born population has a low risk of developing latent tuberculosis: a typical prevalence in this case would amount to only 5 in 100 people. In this case the absolute reduction in number of cases with treatment would be only 2 per 100 people [24]; the difference of 33 per 100 for refugees or immigrants compared to 2 per 100 for high-income country born reflects a difference in baseline rates alone.

### Item generation

Items were generated using three methods. First, existing checklists for applicability, transferability and external validity were assessed for factors related to judging likely differences in relative effects (Additional file 1: Appendix 1). Second, factors associated with subgroup analyses across PROGRESS-Plus were assessed in a systematic review of methods for assessing effects on health equity [25]. Third, practitioners and managers were interviewed using convergent interviewing [26] to identify factors associated with success or failure of program implementation in a vulnerable population [27].

### Item reduction, questionnaire format, scaling, face validity

One author (VW) developed a draft algorithm using the above items. Items were phrased using wording from previously published checklists where possible. Dichotomous yes/no categories were chosen for the responses for two reasons: 1) a “don’t know” category was considered unhelpful in determining likelihood; and 2) the ability of respondents to discriminate more finely than yes/no was uncertain. The draft algorithm was refined with two other authors (PT and GW). The face and conceptual validity was tested by asking four clinician methodologists with experience in clinical epidemiology, systematic reviews and health equity to review the items and judge their clarity and their ability to measure the concept of interest, defined as assessing the likelihood of differences in relative or absolute effects across PROGRESS-Plus characteristics[21]. Subsequent pilot-testing persuaded us to restrict attention to differences in relative effects, thereby dropping further consideration of absolute effects.

### Consistency

Inter-rater reliability of the health equity plausibility algorithm was assessed by recruiting methodologists, clinicians and users of systematic reviews to apply the health equity plausibility algorithm to a sample of 10 systematic reviews. Thirty-five respondents (methodologists, clinicians and users of systematic reviews) were recruited by approaching members of the Cochrane Collaboration who attended the 2009 annual Colloquium. Respondents were asked to judge the likelihood of differences for two PROGRESS-Plus items: 1) sex: female vs. male and 2) low socioeconomic status vs. high socioeconomic status, using the survey (Additional file 1: Appendix 2).

Raters were given a summary for each of 10 systematic reviews inclusion criteria and methods for the population, intervention, comparison, outcomes and study designs included (columns 3–7 of Additional file 1: Appendix 3). Raters were not given the results of the systematic reviews.

The health equity plausibility algorithm was presented to raters as a list of three questions on one page, with a checkbox requiring Yes or No answers. Raters were also given examples of how each of the factors might create important differences in the magnitude of relative effects, based on examples from the updated Journal of American Medical Association (JAMA) User’s Guide on applying results to individual patients [28].

Inter-rater agreement was assessed using Fleiss’ Kappa for multiple raters for 60 assessments (10 systematic reviews X 3 questions X 2 PROGRESS-Plus factors) [29]. We also assessed and interpreted the proportion agreement [30].

The ten systematic reviews chosen for the survey were selected based on the following criteria: 1) proven effective and cost-effective interventions identified by the WHO-CHOICE (World Health Organization CHoosing Interventions that are Cost-Effective) initiative [31]; 2) representation of different types of intervention using the categories defined by the Disease Control Priorities Project of: i) Population-based primary prevention; ii) Personal interventions and iii) Policy instruments [32]; 3) included in the top ten causes of the global burden of disease projected for 2030 [33]; 4) availability of a systematic review with greater than five included studies including a diversity of settings and populations (a meta-analysis was not required since differences across populations can be assessed without a meta-analysis). Candidate reviews were identified by searching MEDLINE and the Cochrane Library by VW. The ten reviews were chosen by discussion of two reviewers (VW, PT) based on the above criteria. They included nine Cochrane reviews and one non-Cochrane review. The attribute of being Cochrane or non-Cochrane reviews was not a criterion for selection since we did not feel there would be important differences in how systematic reviews approach the issue of differences across subgroups based on this attribute.

### Construct validity

The ideal test of construct validity of this health equity plausibility algorithm is comparison with the criterion of whether there are real differences in effects by sex and socioeconomic status, irrespective of whether the instruments (systematic reviews and underlying studies) are able to detect these differences. Comparison to this criterion would be considered concurrent validity. However, since we cannot know the real intervention effects, we used a proxy by considering the results and discussion of the systematic reviews as an indicator of real differences in effects and compared this with the health equity plausibility algorithm to assess construct validity. We extracted these details from the discussion sections of each systematic review by assessing any mention of differences across PROGRESS Plus factors, whether they were supported by evidence or not (e.g. hypothesized differences not supported by evidence were included). We recognize that systematic reviews and underlying primary studies may not be designed or powered to detect such differences (column 2 of Additional file 1: Appendix 3). Therefore, the construct validity of the health equity plausibility algorithm was assessed by comparing whether raters’ assessment of likelihood of important differences in effects were concordant with the discussion of applicability and generalizability by the authors of the systematic reviews across sex and socioeconomic status. Only those comparisons where 70% of raters gave the same judgment were compared with the systematic review conclusions about applicability and generalizability. This cut-off of 70% was chosen based on use of this criterion for consensus recommendations [34].

## Results

### Item generation

A draft health equity plausibility algorithm was developed by three authors (VW,GW,PT) that consisted of four yes/no items: 1) differences in implementation factors across PROGRESS-Plus (e.g. differences in resources); 2) likelihood of variations in the delivery of the intervention across PROGRESS-Plus (e.g. because of poor acceptability, inappropriate literacy level); 3) different mechanism of action of the intervention across PROGRESS-Plus; and 4) differences in expected absolute effects because of higher risk or prevalence across PROGRESS-Plus.

### Item reduction, questionnaire format, scaling, face validity

Consultation with five content experts resulted in adding an item about possible differences in the comparator that might lead to differences in effectiveness. Differences in comparator was defined as whether the “control” groups differed in the context of the systematic review studies compared to the context to which the results would be applied. A question about absolute effects was removed, since it did not fit with the focus of this checklist to identify likelihood of differences in relative effects across PROGRESS-Plus. The revised health equity plausibility algorithm consisted of three questions (Table 1).

**Table 1:** Health equity plausibility algorithm questions

	
**Question 1:**	Are there differences in patient/community/ population characteristics (e.g. underlying pathophysiology, comorbidities, patient attitudes, etc.) that are likely to create important differences in the magnitude of relative effect of the intervention versus the control for the outcome of interest?
**Question 2:**	Are there differences in the way that the intervention is delivered (e.g. provider compliance, provider skill, technical resources, availability of drugs/treatments) that are likely to create important differences in the magnitude of the relative effect of the intervention versus the control for the outcome of interest?
**Question 3:**	Are there differences in the comparator across patient, community or population that are likely to create important differences in magnitude of relative effects?

### Inter-rater consistency

Ten systematic reviews were selected for the field test (Additional file 1: Appendix 3): 1) mass media to promote HIV testing [35]; 2) Population level tobacco control [36]; 3) psychological therapy for post-traumatic stress disorder [37]; 4) first-line anti-hypertensive drugs for people with hypertension [38]; 5) surgical interventions for age-related cataract [39]; 6) vaccines for measles, mumps and rubella [40]; 7) antidepressants vs. placebo in primary care [41]; 8) artemisinin-based combination therapy for uncomplicated malaria [42]; 9) primary safety belt laws [43]; and 10) handwashing for the prevention of diarrhea episodes [44].

Of 43 people contacted, 35 filled out the questionnaire (participation rate= 81%). The 35 respondents represented a mix of users, methodologists and clinicians with a median of 7 years of experience using or conducting systematic reviews and diverse clinical experience (e.g. public health, musculoskeletal, dermatology, cancer, infectious disease) (Table 2). No questions were skipped.

**Table 2:** Characteristics of 35 raters who assessed health equity plausibility

**Experience with systematic reviews**	**User-6**
	**Methodologist-17**
	**Clinician-12**
Years of experience	Median: 7
	Range : 2-15
Area of research/expertise	Public health-8
	Musculoskeletal-7
	Dermatology-1
	Child health-1
	Methods-9
	Family medicine-5
	Infectious disease-2
	Reproductive health-1
	Cancer-1

For the decision about whether important differences existed across sex (e.g. between males and females), the proportion agreement was 66% for patient characteristics, 54% for intervention delivery, and 51% for comparator. Across socioeconomic status, the proportion agreement was 66% for patient characteristics, 79% for intervention delivery, and 59% for comparator. The Fleiss kappa for multiple raters ranged from −0.001 to 0.199 (Table 3 and 4). A kappa of less than 0.2 is considered slight agreement [45], with kappa of 0.2<k<0.4 considered fair; 0.4<k<0.6 moderate, 0:6<k<0.8 substantial and k>0.8 almost perfect agreement.

**Table 3:** Sex/Gender: Health equity plausibility ratings for each question, across 10 systematic reviews

	***Sex: Proportion judging important differences exist across sex***			
Systematic review	*Question 1: Patient differences*	*Question 2: Delivery of intervention*	*Question 3: Comparator*	*Description in systematic review*
**INTER-RATER AGREEMENT on Q1 ≥70%**				
Mass media for HIV testing	96%	70%	57%	Sex differences not analyzed or discussed
Antidepressants for depression in primary care	92%	67%	50%	Sex not discussed or analyzed
Vaccines for MMR in children	8%	17%	25%	Sex not discussed or analyzed.
Primary safety belt laws	83%	67%	33%	Men have higher uptake of seatbelts
Psychological therapy for PTSD	83%	83%	52%	Studies including only females, all of whom had been assaulted, produced more positive results than the overall results.
**INTER-RATER AGREEMENT on Q1 <70%**				
Population tobacco control	70%	48%	48%	No differences found across sex
First line antihypertensives	65%	48%	43%	Females represented 45% of population. No subgroup analyses conducted on sex
Surgery for age-related cataract	67%	67%	67%	Sex not discussed
Hand washing for diarrhoea	67%	50%	50%	Analyses were age and sex adjusted, differences not discussed
ACT for malaria	33%	33%	25%	Sex not discussed
Fleiss Kappa	**0.199**	**0.068**	**0.005**	

**Notes**: PTSD: Post-traumatic stress disorder; SES: socioeconomic status; MMR: Measles, mumps and rubella vaccine.

**Table 4:** Socioeconomic status (SES): Proportion of respondents judging important differences exist for each question, across 10 systematic reviews

	***Proportion of respondents judging important differences exist across SES***				
	*Average rating*	*Question 1: Patient differences*	*Question 2: Delivery of intervention*	*Question 3: Comparator*	*Description in systematic review*
**INTER-RATER AGREEMENT on Q1 ≥70%**					
Mass media for HIV testing	87%	100%	83%	78%	Radio and television interventions can be used in literate and non-literate communities; therefore applicable to LMIC
Antidepressants for depression in primary care	84%	92%	92%	67%	SES not discussed
Population tobacco control	84%	91%	74%	87%	Price increases are more effective in low-income populations. Smoking restrictions: no SES differences
Hand washing for preventing diarrhoea	89%	83%	92%	92%	SES not discussed
**INTER-RATER AGREEMENT on Q1 <70%**					
Surgery for age-related cataract	86%	75%	100%	83%	In developing countries, access to expensive machines, volume of surgeries and skill of surgeons may be lower
Psychological therapy for PTSD	75%	78%	91%	57%	SES not discussed
ACT for malaria	72%	75%	92%	50%	SES not discussed
Primary safety belt laws	72%	58%	100%	58%	More effective for lower use groups (e.g. African-American and Hispanic in USA)
First line anti-hypertensives	67%	65%	83%	52%	SES differences not assessed.
Vaccines MMR in children	67%	50%	75%	75%	SES not discussed. “effectiveness demonstrated world-wide”
**Fleiss Kappa**		**−0.001**	**0.105**	**0.04**	

As part of the survey, respondents were asked to explain the reasoning behind their answers (Table 5). The most common reasons for the answers were based on theory (n=10 raters) and personal experience (n=11). Other reasons for ratings were empirical data (n= 3) and “guesses” (n=4) (8). Respondents stated that other information would have been useful to complete the task, including more information about the interventions and outcomes, clarity on the comparator question and details on the size of difference considered important. Almost one third (10/35) of raters endorsed the importance of considering differences across PROGRESS-Plus in the design of systematic reviews (n=10 people).

**Table 5:** Comments and reactions to making health equity plausibility judgments

**Reason for answer**	**theory-10; personal experience- 11; empirical data-3 guesses-4;**
Other information needed	more intervention specific information and data
	how big are the differences being sought?
	how does comparator overlap with intervention delivery
	was information available from trials?
	consider including community cluster trials
	Need information on how intervention was delivered
General comments	Important to consider these issues in design of SR-5
	Difficult- 4;
	Interesting to consider these issues-5;
	Subjective-6;
	Why only gender and SES- need to consider other factors (e.g. sexual orientation)- 2
	Country differences are important- e.g. if universal drug coverage is available-1
	Accessibility of drugs is less of an issue-1
	Ask questions about heterogeneity-1
	Intervention delivery is important for understanding
	Easy-1

### Construct validity

Our proxy for a “gold standard,” namely a discussion of differences in effect across sex or socioeconomic status appearing in the conclusions of systematic reviews is presented in Additional file 1: Appendix 3, column 1. Each of the ten systematic reviews discussed plausibility of different effects for at least one of three factors: patient characteristics, intervention delivery or comparator. For example, the review of primary safety belt laws discussed greater effects in low use groups, including men, Hispanics and African-Americans. The tobacco control review assessed and found no differences across sex. It is important to note that some of the systematic reviews mentioned differences across PROGRESS-Plus factors, even though they were not supported by evidence. For example, the mass media review mentioned differences between literate and non-literate populations, but this interpretation was not supported by their data no part of any subgroup analyses.

Thirty-two assessments out of 60 (53%) attained greater than 70% agreement between raters (bolded text in Additional file 1: Appendix 4). Of those assessments, 28/31 agreed that differences were likely; 3/31 agreed that difference in effects was unlikely.

The type of intervention did not seem to be associated with agreement. Of those systematic reviews with greater than 70% agreement on the first question on patient characteristics, two were pharmacologic interventions (vaccines and antidepressants) and three were behavioural interventions (PTSD, safety belt laws and mass media) (Table 5).

### Agreement with conclusions of systematic reviews

Seventeen of the assessments with greater than 70% inter-rater agreement (17/32; 53%) were concordant with our proxy for construct validity (the judgments of applicability described by the authors of the systematic reviews). For example, differences in effects due to patient characteristics (question 1) was judged likely for population tobacco control across socioeconomic status, which is supported by the conclusions of the systematic review that state that price control is more effective for low income populations [7].

Eight of the assessments (8/32; 25%) which reached greater than 70% agreement between raters were not consistent with the judgments of the authors of the systematic reviews.

Seven out of 32 assessments (22%) were classified as agreement “Not Known” since the systematic reviews did not discuss plausible or measured differences. For example, 91% of raters judged that important differences were likely across SES due to delivery factors for PTSD, but this was not discussed in the systematic review.

## Discussion

The three questions used for this study were derived from questions which are given to users of systematic reviews to make judgments about clinical practice implications, such as in the JAMA User’s Guide [28]. Although a high proportion of participants rated differences likely across socioeconomic status and sex, the Fleiss kappa indicated low to no agreement beyond chance between raters. Low kappa values in the presence of high agreement has been called one of the paradoxes of kappa [30,45]. Low kappa values may also indicate that there was low contrast in the systematic reviews or the raters, or that some systematic reviews were easier to agree on than others [46]. The low kappa in this study suggest that when making judgments about likely differences in effects, there is a need to have a deep understanding of the content area to make these ratings, and may require the involvement of multiple stakeholders. This has implications for the design of systematic review author teams and advisory boards, as well as for how to form panels of people who make judgments about applicability for the purpose of guidelines or policy decisions.

Secondly, the low kappa observed may be due to multi-component questions covering several factors, and potential confusion of access to health care, prognostic factors and treatment-covariate interactions. Since these three items were derived from questions recommended to users of systematic reviews, this study suggests a need to further refine these questions to improve understanding and increase inter-rater agreement beyond chance.

The strengths of this study are that it followed established steps in developing a checklist of item generation, pilot-testing and assessed consistency and construct validity in a field study. We selected systematic reviews for the field study based on predetermined criteria to maximize diversity of types of interventions (personal and population level) and disease areas with greater than five included studies including a diversity of settings and populations. These ten systematic reviews also included a mix of plausibility of different effects across the three questions.

This study is subject to some limitations. Firstly, the high proportion of “yes” answers may have been due to the way the questions were framed. Secondly, the rationale for affirmative responses was assessed by self-report on a questionnaire, and few details were provided by raters regarding how theory, data or personal experience was used to make these judgments. Thus, we could not assess whether differences were due to some of the reasons that we expected such as the type of study design (randomized vs. observational) or type of comparator (placebo vs. control). Also, given that personal experience was the most common reason for ratings, the raters individual characteristics may be important (e.g. whether they are women, low income, immigrants), and this could be explored in future studies. Both of these limitations could be assessed in further detail by cognitive interviewing using think out loud protocols to assess how respondents interpreted the questions and could be used to improve the questions [47]. Another possible limitation is the use of only two categories (yes/no), and the omission of a “don’t know” category. Raters may have resorted to personal value judgments or random guesses when forced to choose between “yes” and “no”. This might be addressed in future research by using more categories to reflect strong agreement to strong disagreement or by using a visual analogue scale; either of these methods would allow an assessment of confidence of raters in the importance of the difference. Finally, we recognize that our proxy for construct validity yielded unsatisfactory results. We suggest that this indicates a need for improved consideration and reporting of applicability in systematic reviews, particularly for populations to whom the results are likely to be applied.

### Conclusions

This study reinforces the need to develop, evaluate and promote structured methods for considering applicability to relevant populations in systematic reviews. The low kappa suggests a need for a depth of content expertise and stakeholders on systematic review author teams in making such decisions. The results also suggest a need to improve the clarity of questions intended to help users of systematic reviews make applicability judgments. Planning and design of primary research studies and trials needs to take into account whether there are expected and plausible differences across population groups such as sex using appropriate methods such as stratification and pre-planned subgroup analyses. The Campbell and Cochrane Equity Methods Group is conducting methodological research on methods such as the use of logic models, subgroup analysis, applicability judgments and process evaluation as tools for assessing the plausibility of effects on health equity [48].

## Competing interests

Two members of the author team are members of the Campbell and Cochrane Equity Methods Group which conducts methodological research on assessing and reporting effects on health equity in systematic reviews (PT and VW).

## Authors’ contributions

PT and VW had the idea for the study. All authors contributed to the design of the study. VW collected and analyzed data for the study. All authors contributed to writing and revising the manuscript and approved the final version.

## Pre-publication history

The pre-publication history for this paper can be accessed here:

http://www.biomedcentral.com/1471-2288/12/187/prepub

## Supplementary Material

Additional file 1 **Appendix 1.** Applicability and transferability checklists [49-60]. **Appendix 2** Sample equity plausibility algorithm survey provided to raters. **Appendix 3** Characteristics of systematic reviews chosen for testing the equity plausibility algorithm [61]. **Appendix 4** Agreement of equity plausibility ratings between raters for each question and PROGRESS factor, across 10 systematic reviews. Click here for file
