# Supplementary material for: Systematic reviews need to consider applicability to disadvantaged populations: inter-rater agreement for a health equity plausibility algorithm
Source: BMC Med Res Methodol. 2012 Dec 19;12:187. doi: 10.1186/1471-2288-12-187 (PMC3552943; doi:10.1186/1471-2288-12-187)
Supplement: Additional file 1 — Appendix 1. Applicability and transferability checklists [49-60]. Appendix 2 Sample equity plausibility algorithm survey provided to raters. Appendix 3 Characteristics of systematic reviews chosen for testing the equity plausibility algorithm [61]. Appendix 4 Agreement of equity plausibility ratings between raters for each question and PROGRESS factor, across 10 systematic reviews. [file 1471-2288-12-187-S1.doc]

Appendix 1: Applicability and transferability checklists

| **Authors** | **Applicability questions** |
| --- | --- |
| Dans 1998, User’s Guides to the Medical Literature [23] | (1) Are there pathophysiologic differences in the illness under study that may lead to a diminished treatment response?  (2) Are there patient differences that may diminish the treatment response?  (3) Are there important differences in patient compliance that may diminish the treatment response?  (4) Are there important differences in provider compliance that may diminish the treatment response?  (5) Do my patients have comorbid conditions that significantly alter the potential benefits and risks of the treatment?  (6) Are there important differences in untreated patients’ risk of adverse outcomes that might alter the efficiency of treatment? |
| Glasziou 1998 [49] | Is my patient **SO** different that results do not apply?  Is treatment feasible in my setting?  What are likely benefits and harms?  How will my patients’ values influence the decision? |
| Glasziou 2000 [50] | **Applicability defined as**: applying results from studies to individual  What are predicted absolute risk reductions?  Do the benefits outweigh the harms- absolute + net benefits and costs, strength of preferences  **Transferability defined as**: applying results to wider population- infer/predict /project  Benefits and harms  Variations in treatment effect (e.g. subgroups analyses)  How does treatment effect vary with baseline risk? |
| Briss 2000 Community guide [51] | Define target populations and setting  Assess whether target population included in studies  Assess whether population in included studies is representative of target population  Judgment about whether intervention works better or worse |
| Rothwell 2005 [52] | Setting of trial  Selection of patients  Characteristics of patients  Differences between trials and routine clinical care  Outcome measures and follow-up  Adverse effects of treatment |
| Wang 2005 [53] | If implemented, will effectiveness be the same: Baseline prevalence, characteristics of population, capacity to implement  Whether the intervention could be implemented: political environment, social acceptability, cultural adaptability, resource implications, educational level, organizational structure |
| Green and Glasgow 2006 [54] | Reach and representativeness  Program or policy implementation and adaptation  Outcomes for decision making (e.g. moderator effects, costs)  Maintenance and institutionalization |
| AHRQ methods guide 2007 | Population, Intervention intensity and quality, Comparator (and dosing), Outcomes, Timing of follow-up |
| CONSORT 2001 [55] | External validity, also called generalizability or applicability, is the extent to which the results of a study can be generalized to other circumstances (181).  Patient characteristics (e.g. age, sex, severity of disease, and comorbid conditions?)  Intervention (e.g. drugs within a class of similar drugs, different dosage, timing and route of administration)  Comparison (e.g. different concomitant therapies)  Setting (e.g. primary, secondary, and tertiary levels of care)  Outcomes (e.g. effect on related outcomes that were not assessed in the trial, and the importance of length of follow-up and duration of treatment) |
| Cochrane Handbook 2008 [56] | Biologic variation  Variation in context and culture  Variation in adherence  Variation in values and preferences |
| Lavis [57] | Could it work? structural elements  Will it work (or what would it take to make it work)? E.g. power dynamics, other priorities/realities  Is it worth it? Balance of benefits and harms worth the incremental costs? |
| Dans 2007 [58] | Do the public health recommendations in the guidelines address a priority problem for disadvantaged populations?  Is there a reason to anticipate different effects of intervention in disadvantaged and privileged populations?  Are the effects of the intervention valued differently by disadvantaged compared with privileged populations?  Is specific attention given to minimizing barriers to implementation in disadvantaged populations?  Do plans for assessing the impact of the recommendations include disadvantaged populations? |
| PRISMA 2008 [59] | *“Item 24: Summary of evidence*  Summarise the main findings, including the strength of evidence for each main outcome; consider their relevance to key groups (such as healthcare providers, users, and policy makers)… Although there is no standard way to assess applicability simultaneously to different audiences, some systems do exist.” |
| EUROTHINE – judging transferability to the Netherlands setting [60] | -method, internal validity,  -observed effects in lower SES groups  -potential for effect on health inequalities,  -relationship to other programs,  -evidence needed for evaluation  - experiences with implementation,  -possibilities to adjust intervention to the Dutch situation  -mechanisms that explain observed effect,  -differences between Netherlands and study population in relevant factors,  -observed variations in effectiveness according to population, place, period  -estimation of effectiveness in the Netherlands |
| Dans 2008, JAMA Users Guides [28] | A. Can I apply the results to my patients?  1. Have biologic factors that might modify the treatment response been excluded? (across sex, comorbidities, race, age and pathology)  2. Can the patients comply with treatment requirements?  3. Can the health care providers comply with treatment requirements?  B. Are the benefits worth the risks and costs? |

Appendix 2: Sample equity plausibility algorithm survey provided to raters

**Task: Please think about the question described in PICO format above. Do you think there are likely to be important differences in the magnitude of relative effects across: 1)women vs. men or 2)poorer vs. richer people (low socioeconomic status- SES)?**

**About you:** Are you practitioner, consumer, statistician: please describe? _____________

How many years experience with Cochrane and evidence-based medicine?____________

What is your area of research/expertise?________________________

| **Question** | **Surgical interventions for age-related cataract** | **Vaccines for measles, mumps and rubella in children** | **Antidepressants versus placebo for depression in primary care** | **Artemisinin-based combination therapy for treating uncomplicated malaria** | **Primary safety belt laws to reduce fatal and nonfatal injuries in vehicle crashes** | **Hand washing for preventing diarrhoea** |
| --- | --- | --- | --- | --- | --- | --- |
| 1.Are there differences in **patient/community/ population characteristics** (e.g. underlying pathophysiology, comorbidities, patient attitudes, etc.) that are likely to create **important difference**s in the magnitude of relative effect of the intervention versus the control for the outcome of interest?  [e.g. pathophysiology: aspirin has greater relative effect on stroke prevention in women than men;  e.g. co-morbidities: poor people have higher co-morbidities than richer people;  e.g. patient attitudes: women may have different preferences for type of care than men] | Gender/Sex | Gender/Sex | Gender/Sex | Gender/Sex | Gender/Sex | Gender/Sex |
|  | **Y/N** | **Y/N** | **Y/N** | **Y/N** | **Y/N** | **Y/N** |
|  | Low SES | Low SES | Low SES | Low SES | Low SES | Low SES |
|  | **Y/N** | **Y/N** | **Y/N** | **Y/N** | **Y/N** | **Y/N** |
| 2.Are there differences in the way that the **intervention is delivered** (e.g. provider compliance, provider skill, technical resources, availability of treatments) that are likely to create **important differences i**n the magnitude of the relative effect of the intervention versus the control for the outcome of interest?  e.g. surgeon skill in hospitals with less surgeries may be lower than in clinical trials | Gender/sex | Gender/sex | Gender/sex | Gender/sex | Gender/sex | Gender/sex |
|  | **Y/N** | **Y/N** | **Y/N** | **Y/N** | **Y/N** | **Y/N** |
|  | Low SES | Low SES | Low SES | Low SES | Low SES | Low SES |
|  | **Y/N** | **Y/N** | **Y/N** | **Y/N** | **Y/N** | **Y/N** |
| 3.Are there differences in the **comparator** across patient, community or population that are likely to create **important differences** in magnitude of relative effects?  e.g. poor people may live in an environment with poorer water quality | Gender/sex | Gender/sex | Gender/sex | Gender/sex | Gender/sex | Gender/sex |
|  | **Y/N** | **Y/N** | **Y/N** | **Y/N** | **Y/N** | **Y/N** |
|  | Low SES | Low SES | Low SES | Low SES | Low SES | Low SES |
|  | **Y/N** | **Y/N** | **Y/N** | **Y/N** | **Y/N** | **Y/N** |
| 4.What is your reason for your answer? Theory, personal experience, other data?  Please feel free to use more space and to separate gender/sex and SES | Gender:  SES: |  |  |  |  |  |

Appendix 3: Characteristics of systematic reviews chosen for testing the equity plausibility algorithm

|  | **Difference across sex or SES? (likely or confirmed)** | **Population** | **Intervention** | **Comparison** | **Outcome** | **Eligible study designs** | **Studies included** | **Applicability judged by authors** | **Implementation** | **Setting** | **Results** |
| --- | --- | --- | --- | --- | --- | --- | --- | --- | --- | --- | --- |
| Population tobacco control interventions [7] | Sex: No difference found  SES: Yes, for price control | People smoking, at risk of smoking, exposed to ETS or general population, | Population level tobacco control (remove subsidies on production, restricting trade, restrict advertising, health warning labels, restricting smoking in public places) | Usual exposure to smoking cessation and prevention | Changes in smoking behavior, sales for people with different demographic or socioeconomic characteristics | Any design | 84 studies | Increasing the price of tobacco products is more effective in low-income populations.  Smoking restrictions in workplaces and public places are not more effective among more advantaged groups | Studies often do not often describe contextual factors or co-interventions which may affect results | Over half of studies conducted in the USA and six in the UK.  Priority to assess effects in other country settings and contexts | Most compelling evidence on favouring the less well-off is increased price of tobacco |
| Primary safety belt laws to reduce fatal and nonfatal injuries in vehicle crashes [43] | Patient characteristics: more effective for lower use groups (e.g. African-American, Hispanic, male)  Delivery: no difference in enforcement across race/ethnicity  Comparator: prevalence of wearing seatbelt is lower for males, rural people, African-American and Hispanic | General population | Primary enforcement laws allow a police officer to stop  a motorist solely for not wearing a safety belt | No safety belt laws | Safety belt  use, crash-related morbidity and mortality | Any evaluations of safety belt laws | 13 studies of primary vs. secondary enforcement | Seatbelt use increased more in African-Americans and Hispanics than whites; also higher uptake by rural people and males | Studies show that enforcement is similar across race and ethnicity; public opposition may be a barrier to implementation | Diverse settings and target groups; | Fatalities decreased by 8% with primary compared to secondary laws |
| Mass media to promote HIV testing [35] | Sex: Not described  SES: No- “applicable in non-literate” | General public, target groups (e.g. sex workers, drug users, bisexual, pregnant women, adolescents) | Specific or general mass media campaigns, targeted at population or specific groups that aim to increase counseling and testing, including radio, TV, print, film, billboards, folk media. | Control group or pre-intervention levels, In relation to relative increase of HIV testing over time | HIV testing by the general population or specific target populations, HIV seroprevalence | RCT, CCT, interrupt-ed time series | 2 RCTs, 3 CCT, 9 ITS | “Radio and television interventions can be used in literate and non-literate communities; therefore they are applicable to LMIC” | Quality of media campaigns was difficult to assess- e.g. duration, intensity, adherence to social marketing principles | Did not find any studies in high risk groups or epidemic countries, or comparisons of different methods, or assessment of cost-effectiveness | Effective in short term (slope change 5.5 SD units (95% CI 2.4-8.6) |
| Psychological treatment for post-traumatic stress disorder (PTSD) [37] | Sex: Yes, works better in women  SES: not described | Adults suffering from traumatic stress symptoms for three months or more | Trauma-focused or group cognitive behavioural therapy, stress management, supportive therapies, psychodynamic therapies | Control, placebo, waiting list, usual care, alternative psychological therapies, Usual care allowed | Severity of clinician-rated or self-reported traumatic stress symptoms | RCTs | 33 RCTs | Studies including only females, all of whom had been assaulted, produced more positive results than the overall results. | Diversity of clinical interventions | Issues with the control group since it is difficult to blind participants and therapists and to have a “placebo” for psychological therapy | Improvement in clinician assessed PTSD symptoms immediately after treatment (large effect size as assessed with SMD) |
| First-line drugs for hypertension [38] | SEX: No, females represented 45% of population  SES: Not described | Adult with baseline resting BP => 140 mm Hg systolic or diastolic BP =>90 mm Hg. >70% patients high BP | First-line anti-hypertensive therapy: thiazide diuretics, beta blockers, calcium channel blockers, angiotensin converting enzyme (ACE) inhibitors, angiotensin II receptor antagonists or alpha adrenergic blockers | Must be placebo, or an untreated control. Other drugs allowed if taken by <50% of patients | Mortality, stroke, coronary heart disease, cardiovascular events | RCT >1 year | 24 RCTs, 58,040 patients included | Females represented 45% of population, 10/24 trials reported ethnicity of 0-80% african-american), 72% of people studied were primary-prevention | 72% of participants were primary prevention.  Results for secondary prevention were less robust | Most participants were recruited from Western industrialized countries (15% USA, 66% Europe, 7% Australia) | Low dose thiazides decrease coronary heart disease (RR 0.72, 95% CI 0.61 to 0.84); but high dose thiazides did not (RR 1.01, 95% CI 0.85 to 1.20) |
| Surgical interventions for age-related cataract [39] | Patient differences: not discussed  Delivery: expensive machines and highly skilled surgeons needed for most effective method; difficult in LMIC  Comparator: higher prevalence of uncontrolled cataracts in LMIC | people with age-related cataract | different surgical interventions for age-related cataract | Different types of surgery or waitlists | Visual acuity | RCTs | 17 RCTs, 9627 people | Need more studies in developing countries where access to expensive machines, volume of surgeries and skill of surgeons may be lower | Phacoemulsification requires expensive machine (20,000 pounds), highly skilled surgeons  Barriers to surgery in LMIC | 8 studies in Europe, 2 in the far east and 6 in India, 1 in Africa  Higher prevalence and less control in LMIC | Pharmacoemulsification gives the best outcomes, but will only be accessible in poorer countries if the cost decreases |
| Vaccines for measles, mumps and rubella in children [61] | Patient characteristics: not described, but implications for practice state that effectiveness demonstrated world-wide  Delivery: not described, but implications for practice state that effectiveness demonstrated world-wide  Comparator: not described | healthy individuals up to 15 years of age | Vaccination with any combined MMR vaccine given independently,  in any dose, preparation or time schedule | Do-nothing or placebo. | Clinical cases: measles, mumps or rubella | All comparative prospective or retrospective studies | 5 RCTs, 1 CCT, 14 cohort studies, 5 case-control studies, 3 time series trials, 1 cross-over, 1 ecological trial, 1 self-controlled case series trial | External validity of included studies was low | inadequate description of populations, response rates, vaccine content and exposure | Setting of studies was not described in SR | Limited evidence of safety of MMR, lack of field studies on effectiveness of MMR |
| Antidepressants versus placebo for depression in primary care [41] | Patient characteristics: not discussed,  Delivery: not discussed, primary care practitioners may be more likely to change therapy if adverse effects occur  Comparator: not discussed | patients (under the age of 65 years) with depression in primary care | tricyclic antidepressants (TCAs) or selective serotonin reuptake  inhibitors (SSRIs | Placebo | Depression symptoms | RCTs | 14 RCTs | Results apply to patients with major depressive disorder and heterogeneous depression; NNT of 6-16 are comparable to other treatments in primary care | All studies gave a clear description of the treatments and concurrent therapies | 9/14 studies had “representative samples” (ie, outpatient, general practice settings ) | Benefit of TCA and SSRI (NNT of 6-16), consistent across subgroup and sensitivity analyses for quality, setting and type of depression |
| Artemisinin-based combination therapy for treating  uncomplicated malaria [42] | Patient characteristics: endemicity and resistance affect effectiveness  Delivery: not discussed  Comparator: not discussed | People with uncomplicated *P. falciparum*malaria | Artemisinin-based Combination  Therapy (ACT) | Other antimalarials | Treatment failure | RCTs | 50 RCTs | Interpret summary statistics with caution since there are changing patterns of resistance which vary with place and time | Very young children and pregnant women were excluded in these trials | 31 RCTs in Africa, 17 n Asia, in south America, 1 in Oceania; resistance and endemicity are important | All five ACTS met the WHO criteria for less than 10% failure rate |
| Hand washing for preventing diarrhea [44] | Patient characteristics: not described (though analyses were age and gender adjusted)  Delivery: need long-term studies of effectiveness in LMIC (less monitoring)  Comparator: availability of water and materials in LMIC | General population in institutions (e.g. day-care centres), households, or communities | interventions to promote hand washing e.g., small group discussions, multimedia  communication campaigns (tv,radio, leaflets, comic books, songs, slide shows, use of T-shirts and badges, pictorial stories, dramas, and games.) | No intervention to promote handwashing | Diarrhoeal episodes in children and adults | RCTs | 14 RCTs | Uncertain if effectiveness will be maintained if scaled up to larger population with less monitoring and over longer time period  Results cannot be generalized to all ages (all children were less than 15 and most less than 7 yr years) | Handwashing may require infrastructure, cultural, behavioural changes and resources; assessed hand washing method, style, materials provided, water availability | 8 in high-income countries, 5 in LMIC, 1 in HIV/AIDS patients | 39% reduction in diarrhea episodes in children in HIC, 32% reduction in LMIC |

Appendix 4: Agreement of equity plausibility ratings between raters for each question and PROGRESS factor, across 10 systematic reviews

|  | ***Question 1: Patient differences***  ***Agreement between raters***  ***(Proportion of yes answers)*** | | ***Question 2: Delivery of intervention***  ***Agreement between raters***  ***(Proportion of yes answers)*** | | ***Question 3: Comparator***  ***Agreement between raters***  ***(Proportion of yes answers)*** | |
| --- | --- | --- | --- | --- | --- | --- |
| Systematic review | Sex | SES | **Sex** | SES | Sex | SES |
| Mass media for HIV testing | **0.91^NK^**  **(96%)** | **1 ^x^**  **(100%)** | **0.56 ^NK^**  **(70%)** | **0.70 ^x^**  **(83%)** | 0.49  (57%) | **0.64 ^x^**  **(78%)** |
| Population tobacco control | **0.56 ^x^**  **(70%)** | **0.83 ^y^**  **(91%)** | 0.48  (48%) | **0.60 ^y^**  **(74%)** | 0.48  (48%) | **0.76^y^**  **(87%)** |
| Psychological therapy for PTSD | **0.70 ^y^**  **(83%)** | **0.64 ^x^**  **(78%)** | **0.70 ^y^**  **(83%)** | **0.83 ^x^**  **(91%)** | 0.48  (52%) | 0.49  (57%) |
| First line antihypertensives | 0.53  (65%) | 0.53  (65%) | 0.48  (48%) | **0.70 ^NK^**  **(83%)** | 0.49  (43%) | 0.48  (52%) |
| Surgery for age-related cataract | 0.52  (67%) | **0.59 ^y^**  **(75%)** | 0.52  (67%) | **1.00 ^y^**  **(100%)** | 0.52  (67%) | **0.70 ^y^**  **(83%)** |
| Vaccines for measles, mumps and rubella in children | **0.83 ^y^**  **(8%)** | 0.45  (50%) | **0.70 ^y^**  **(17%)** | **0.59 ^x^**  **(75%)** | **0.59 ^y^**  **(25%)** | **0.59 ^x^**  **(75%)** |
| Antidepressants versus placebo for depression in primary care | **0.83 ^NK^**  **(92%)** | **0.83 ^NK^**  **(92%)** | 0.52  (67%) | **0.83 ^NK^**  **(92%)** | 0.45  (50%) | 0.52  (67%) |
| Artemisinin-based combination therapy for treating  uncomplicated malaria | 0.52  (33%) | **0.59 ^y^**  **(75%)** | 0.52  (33%) | **0.83 ^y^**  **(92%)** | **0.59 ^y^**  **(25%)** | 0.45  (50%) |
| Primary safety belt laws | **0.70 ^y^**  **(83%)** | 0.47  (58%) | 0.52  (67%) | **1.00 ^y^**  **(100%)** | 0.52  (33%) | 0.47  (58%) |
| Hand washing for preventing diarrhoea | 0.52  (67%) | **0.70 ^NK^**  **(83%)** | 0.45  (50%) | **0.83^NK^**  **(92%)** | 0.45  (50%) | **0.83 ^y^**  **(92%)** |
| Fleiss Kappa | 0.199 | -0.001 | 0.068 | 0.105 | 0.005 | 0.04 |

**Note**: Bolded items indicate greater than 70% agreement between raters. Superscripts indicate whether results agree with the discussion sections of the systematic reviews: NK: not known, Y: agree with discussion section of systematic review, X: disagree with discussion section of systematic review.
